# Cultural transition of international medical graduate residents into family practice in Canada

**DOI:** 10.5116/ijme.570d.6f2c

**Published:** 2016-05-04

**Authors:** Jean A.C. Triscott, Olga Szafran, Earle H. Waugh, Jacqueline M.I. Torti, Martina Barton

**Affiliations:** 1 Department of Family Medicine, University of Alberta, Canada; 2 Department of Family Medicine, University of Calgary, Canada

**Keywords:** Cultural competency, foreign medical graduates, family practice, Canada

## Abstract

**Objectives:**

To identify the perceived strengths that international medical graduate (IMG) family medicine residents possess and the challenges they are perceived to encounter in integrating into Canadian family practice.

**Methods:**

This was a qualitative, exploratory study employing focus groups and interviews with 27 participants - 10 family physicians, 13 health care professionals, and 4 family medicine residents. Focus group/interview questions addressed the strengths that IMGs possess and the challenges they face in becoming culturally competent within the Canadian medico-cultural context. Qualitative data were audiotaped, transcribed, and analyzed thematically.

**Results:**

Participants identified that IMG residents brought multiple strengths to Canadian practice including strong clinical knowledge and experience, high education level, the richness of varied cultural perspectives, and positive personal strengths.  At the same time, IMG residents appeared to experience challenges in the areas of:  (1) communication skills (language nuances, unfamiliar accents, speech volume/tone, eye contact, directness of communication); (2) clinical practice (uncommon diagnoses, lack of familiarity with care of the opposite sex and mental health conditions); (3) learning challenges (limited knowledge of Canada’s health care system, patient-centered care and ethical principles, unfamiliarity with self-directed learning,  unease with receiving feedback); (4) cultural differences (gender roles, gender equality, personal space, boundary issues; and (5) personal struggles.

**Conclusions:**

Residency programs must recognize the challenges that can occur during the cultural transition to Canadian family practice and incorporate medico-cultural education into the curriculum.  IMG residents also need to be aware of cultural differences and be open to different perspectives and new learning.

## Introduction

In Canada, international medical graduates (IMGs) are physicians who completed their medical degree from a non-North American accredited medical school.^1^ They comprise approximately 25% of practicing physicians in Canada.^1^ For many IMGs, the primary route of entry into medical practice is through postgraduate residency training.  The majority of those who are successful in obtaining a residency position do so in family medicine.  IMG family medicine residents are a heterogeneous group of learners with distinct ethnic, religious, and cultural backgrounds.  They come from a variety of countries with differing educational and clinical experiences,^2^as well as differing societal values.  The interaction of different cultural norms and perspectives, and the process of learning and adapting to culture is referred to as cultural transition.^3^^,^^4^  The varying cultural perspectives and experiences that IMG residents possess can be both sources of strength and challenges when transitioning into clinical practice in Canada.  

Current literature on IMGs’ experiences in western cultural settings, and the benefits and challenges thereof, largely fall into four general spheres of experience: medical knowledge and scope of practice; cultural norms and expectations; communication; and individual/personal experiences.  Medical knowledge and scope of practice comprises both clinical education and familiarity with the local health care system.^5^  IMGs often face new types of diseases, different methods of examination and patterns of treatment, as well as new types of medical technology.^6^  Cultural norms and expectations include gender roles in doctor-patient interactions and in interactions with colleagues and preceptors, patient expectations, the doctor-patient relationship, as well as the role and status of the physician within the medical community and general society.^3^^,^^6^ Communication includes both formal and idiomatic communication, in addition to non-verbal communication, between the physician and patient, as well as preceptors and colleagues.^7^  Individual and personal experiences refer to emotional struggles of IMGs such as stress, loneliness, family disruption, as well as discrimination based on race, gender, or nationality.^6^ 

The doctor-patient relationship has been cited as a major source of conflict for IMGs transitioning into new environments.^7^ IMGs may struggle to adjust to a patient-centered relationship where physicians “share power, responsibility, [and] decision-making” with patients.^7^  They may also have difficulty with the more interactive, relationship-centered model of communication, which aims to increase the depth and quality of interaction between physician and patient.^ 7  Another^ recurring theme of cultural exchange on the doctor-patient relationship is the notion that shared beliefs or similarity in culture between patient and physician result in improved patient experience and outcomes.^8^

The transition of IMGs into western medical culture is also influenced by communication.  Several qualitative studies of IMGs working in western countries note that, although many IMGs have considerable training and experience with formal language, informal and region-specific language can limit effective communication.^9^  Non-verbal communication, such as facial expressions and hand movements, are reported as potential barriers for IMGs during their transition.^2^  In addition, when accents are considered too strong or unfamiliar by the patient, this can affect the doctor-patient relationship.^8^^,^^10^

The number of IMGs in residency training programs across Canada has been increasing,^1^ and there is a need to explore and understand the cultural transition in the family practice setting.  An understanding of the strengths and challenges that IMG family medicine residents encounter when transitioning to the Canadian medico-cultural context can facilitate the development of resources for successful transition into practice in Canada. 

The purpose of this study was to gain an understanding of the cultural transition of IMG residents into family practice in Canada.  Specifically, to identify the perceived strengths that IMG residents possess and challenges they encounter as they begin the process of integrating into Canadian family practice, as perceived by themselves and by those who teach and work with them.

## Methods

Study design

This was a qualitative, exploratory study combining focus groups and individual interviews with family physicians, other health care professionals, and family medicine residents.  From a cultural perspective, the focus of this study was on IMG residents who immigrated to Canada and obtained their medical degree from non-North American accredited medical schools.  Canadian citizens who obtained their medical degree abroad and then returned to Canada for residency training were excluded from consideration, as they were deemed to be already acculturated or had previously undergone the transition. 

Focus group methodology was employed to generate insightful and in-depth discussion and to obtain combined perspectives.  In order to accommodate the limited availability of the busy individuals, as well as to include the perspectives of a broad range of health professionals, interviews were also conducted with some participants who could not attend a focus group.  Data collection was conducted during the period July 2012 and November 2014. 

### Study participants and recruitment

The study participants included academic or community-based family physicians who were involved in training IMG residents, allied health care professionals (nurses, quality coordinators, clinic staff, etc.) working within the family practice setting, and family medicine residents (Canadian medical graduates and IMGs).  Academic or community-based family physicians who were themselves IMGs, but who at the time of the study, were involved in training IMG residents, were included.  Family physicians and residents were recruited from the Departments of Family Medicine at the University of Alberta and University of Calgary.  Allied health professionals were recruited from academic family practice teaching sites affiliated with the two departments.

Recruitment letters were mailed or emailed to academic family physicians. Names, addresses, and emails were obtained from the respective department’s website.  If contact information was not available on the website, a recruitment letter was sent to the respective department for forwarding to the academic faculty member.  Community family physicians were also recruited via a recruitment letter which was forwarded to the respective department for mailing or emailing to the physician. Residents were recruited through the respective departments via a recruitment letter emailed to all first year and second year family medicine residents.  A recruitment letter was distributed by the affiliated family practice teaching sites to allied health professionals who worked in the family clinics.  All participants who were interested in taking part in the study were requested to contact the study team and all those who agreed to take part were included. Ethical approval was obtained from the Health Research Ethics Board (Health Panel), University of Alberta, and the Conjoint Health Research Ethics Board, University of Calgary.

### Focus groups and interviews

Participants who agreed to take part in the study and who provided written consent took part in either a focus group or an interview.  In situations when there were scheduling conflicts or when participants were unable to attend the scheduled focus groups, individual interviews were conducted.  One of the co-authors (JT) conducted the focus groups and in-person interviews in both Edmonton and Calgary. Separate focus groups were conducted with academic/community-based family physician teachers, allied health professionals working in the family practice setting, Canadian-graduated family medicine residents, and IMG family medicine residents.  All focus groups and interviews were audiotaped.

Study participants were asked the following two questions related to the cultural integration of IMG family medicine residents:  (1) What are the strengths of IMGs working in the Canadian health care system? and (2) What are the barriers that IMGs face in becoming culturally competent within the Canadian health care system?  According to the interview guide, the questions were open-ended and asked in the same order each time. The interviewer/facilitator had some flexibility in re-phrasing the questions. Several prompts were included under each question to elicit in-depth information and/or stimulate recall.  No time restrictions were placed on the interviews/focus groups and participants were free to speak to any question for as long as they wished.  The perspectives of those who train and work with IMG residents and of IMGs themselves was deemed important in this study so as to develop an understanding of the Canadian medico-cultural norms and values that are expected in this health care setting.

### Data analysis

Audiotaped qualitative data were transcribed verbatim by a professional transcriptionist, then cleaned to remove any identifiable information, and subsequently analyzed for emerging themes.  Thematic analysis was conducted by four of the study investigators (JACT, OS, EW, JT) who independently analyzed each transcript by coding and identifying themes.  The investigators met on a regular basis to discuss individual analysis findings and worked to reach a consensus when categorizing codes.

## Results

In total, 27 participants took part in the study - 10 family physicians, 2 Canadian-graduated residents, 2 IMG residents, and 13 allied health professionals.  A total of 6 focus groups (3 with family physicians, 1 with Canadian-graduated residents, and 3 with health professionals) and 7 individual interviews (4 physicians, 2 IMG residents, 1 allied health staff) were conducted.   Twenty-two participants were female and 5 were male (all 5 males were family physicians).  On average, focus groups were 62 minutes and individual interviews were 53 minutes in duration.

### Strengths of IMG residents

The strengths of IMG residents identified by study participants were along four main themes:  (1) strong clinical/medical knowledge and experience; (2) high education level; (3) richness of cultural perspectives; and (4) positive personal strengths.

#### Strong clinical/medical knowledge and experience

IMG residents were noted to possess strong clinical skills and medical knowledge.  Many were trained and practiced as specialists abroad, with some practicing in more than one country.  Those from tropical countries were knowledgeable about a broad range of infectious diseases and tropical medicine.  It was also noted that many IMGs demonstrated strong physical examination and procedural skills, in addition to quick decision-making skills, and were knowledgeable about various medications and traditional healing methods not common in Canada.

“I think they often have significant experience.  A lot of IMGs are coming from diverse backgrounds where they’ve had significant patient care experience.  They’ve seen a lot of conditions at a different part of the illness spectrum than many Canadian physicians have, so have a better understanding of what those conditions look like untreated. They also often will have very good physical examination skills because in many countries they’re more reliant in that with less access to technology.” (Physician 9)

“…because of the volume of patients that they see, certain specialities are very well trained in terms of their medical knowledge and their hands-on experience compared to us.” (Resident 2)

#### High education level

Participants indicated that many IMG residents were highly educated, with some having multiple academic degrees (MSc, PhD) from their home country.  In addition to medical degrees, some had research training or health policy and administration experience.  As specialists in their home country, many were able to bring the knowledge and expertise of their specialty into family practice in Canada.  It was also noted that Canadian preceptors can often learn a great deal from IMG residents, particularly as it relates to different cultural views and perspectives on health and illness, as well as alternative healing modalities. 

“…we have a fellow right now who I think did quite a bit of cardiology or he was [in] internal [medicine] and specialized in cardiology … he can look at an ECG from across the room and there’s a left bundle there, and oh, might be a bit of an AV block, …I’m like, I think I see a ‘p‘ wave. I can’t see much at all, and he’s got great background in that and so they bring really sometimes very interesting and useful parts of their training to the plate.” (Physician 7)

“I’ve worked with a number of IMGs that were anesthesiologists, one was an infectious disease doctor, one was a surgeon and one was an emerge[ency] doctor back in their home country.” (Resident 1)

#### Richness of cultural perspectives

The diversity of cultural backgrounds of IMGs was considered to be a valuable asset, particularly in multicultural Canada.  Many IMGs speak multiple languages which facilitates communication with culturally diverse patients.  Sharing a common language and a common culture with patients was seen to provide a level of comfort and assist physicians in establishing trust and rapport with patients. 

“And then there’s that little lady who speaks their language that for the first time she’s so excited, she can actually tell them everything she wants them to know. So that’s a huge strength when you have a resident with their same language.” (Allied Health Professional 5)

“…it allows them to identify with other members of their culture…that puts those patients in turn at ease because they’re seeing something that they are used to and being treated in a manner that they’re used to, and they have so many similarities that it’s just easier to identify with somebody.”  (Resident 1)

IMG awareness of different cultural perspectives and practices was also deemed to benefit those colleagues (preceptors, other residents, other health professionals) working alongside IMG residents to understand their own patients from distinct cultural backgrounds.

“The IMGs have helped me understand my patients, as I get to spend time with my colleagues who are from different cultures and countries, I learn about my patients.”  (Physician 3)

IMG residents were also perceived to be compassionate, understanding, and empathetic to minority groups and underserved populations.  Many came from cultures that have high regard and respect for the elderly.  These qualities were regarded as valuable for improving the awareness of marginalized groups in Canada.

“Then I think there is maybe more of a depth of understanding with minorities and more compassion towards them...” (Allied Health Professional 13)

“And just more of an understanding of the pressures to look after your elderly and…the intergenerational interactions which are different than here.” (Allied Health Professional 13)

#### Positive personal strengths

Participants commented that IMG residents were often older than Canadian-graduated residents and thus brought their life experience, knowledge, skills and maturity into clinical practice.  They were described as being determined, confident, and hardworking individuals who were serious about their work and adapted readily to situations.

“…who tend to be older and tend to have families, tend to have children, especially in the family medicine context…they will understand relationships and families…” (Physician 4)

“Any time I ask for volunteers from the resident group, you can be assured that the 12 out of the 80, the 12 that are the IMGs will always volunteer, despite them having families and despite them being exhausted from having to take care of their in-laws who also live with them, they will volunteer.” (Physician 7)

### Challenges faced by IMG residents

The challenges faced by IMG residents were grouped into five theme areas: (1) communication/language; (2) clinical practice challenges; (3) learning challenges; (4) cultural differences; and (5) personal challenges.

#### Communication/language

Communication was a predominant theme identified by physicians and health professionals notably, that IMG residents often struggled with language and communication issues within the Canadian context.  Lack of fluency in the English language and unfamiliar accents were noted to impede communication with patients and staff.  For some, grammar and spelling were also a challenge.

“…they get the scientific thing…but I don’t think they get the emotional feeling, sometimes they miss out on what we’re really trying to say just because of language barriers.” (Allied Health Professional 8)

“They’re nodding but you’re not sure that they’re really comprehending.” (Allied Health Professional 3)

“…you can tell they have very good English but they still don’t have good communication skills, or the communication skills we consider good by Canadian standards.” (Physician 3)

Nuances of the English language, both verbal and non-verbal, were often not readily understood by some IMG residents. This included lack of awareness regarding Canadian norms of speech volume and tone, as well as cultural differences in the use and expression of humor were also noted as challenges. 

“…I think, one of the most difficult ones are the nuances of language, catching on to the errorisms of talk.  In medicine, that’s even more accentuated…the effectiveness of family medicine is highly based on the relationship between the patient and the doctor and the rest of the team the doctor’s working with, and if that relationship is interfered with by misunderstandings of language it makes life difficult and diagnosis difficult and affects everything, down to patient compliance…” (Physician 1)

Interestingly, the IMG residents who took part in this study did not comment on communication and language challenges.  One of the IMG residents did, however, mention that those IMGs trained in non-English speaking countries may be judged as being slow, as they must mentally translate messages into their first language for comprehension and then translate back to English to respond. 

“So the tendencies for them to be perceived as being slow, but the truth is, it’s a whole challenge listening to something, trying to interpret it in the language you are used to, and then coming up with an answer and then being able to put that forth in English, so that gives them the impression that they’re slow, but it’s just that it’s a major challenge having to deal with a language, …that is not their first language, especially if that was how they received their training.  So it’s a major challenge that IMGs face.” (Resident 3)

Participants identified that eye contact was a challenge for those residents from cultures that have strict rules about eye contact between the sexes or from cultures where extended eye contact is interpreted as a challenge to authority. 

“…[some] are so soft-spoken, you combine that with the accent and then they don’t make the good eye contact, it’s a huge challenge to really figure out what they’re saying, both from a colleague perspective or a patient’s perspective.” (Resident 1)

Participants also recognized that IMGs were faced with becoming familiar with copious amounts of medical jargon, use of medical slang, medical abbreviations and acronyms.  Quite often the names of illnesses/diseases also differed from that of other countries.

“But sometimes word use, things like stomach flu which are peculiar to North American culture, other people don’t get influenza in their stomachs.” (Physician 8)

It was also noted that some IMG residents were used to more direct communication styles when dealing with patients which were sometimes judged to be ineffective or offensive in the Canadian context.

“I watched an IMG…he’d be like, you’re fat, that’s why your joints suck, and the patient started to cry because nobody says that stuff here, but he had trained in a different country that was maybe more direct.”  (Resident 1)

#### Clinical practice challenges

Participants remarked that early on in their training in Canada, IMG residents tended to look for diagnoses that were common in other parts of the world, but were rare in Canada, and they appeared to focus on coming up with a quick diagnosis and clinical decision.  They also seemed to be unaccustomed to patient visits for minor issues.

“…they’re always looking for those rare things that in Canada just don’t happen…they would treat every kid with viral gastroenteritis with not just [any] kind of antibiotic, but like, hardcore long term IV antibiotics, and it’s just because where they’re from, and they’d also look for parasites too, which we don’t see...” (Resident 1)

“…I’ve worked with a number of IMGs that find it really frustrating that somebody will come in for a simple cold or a cough ‘cause that’s certainly not what they would have seen before.” (Resident 1)

Some IMG residents were noted to lack knowledge and training in caring for individuals of the opposite sex.  In some cultures, traditional norms related to gender interactions dictate that female physicians care for female patients and male physicians for male patients.  Residents trained in these systems tended to lack anatomical knowledge of the human body of the opposite sex and were unfamiliar with gender specific illnesses/conditions.

“I can recall one instance where a resident who was quite experienced in his own discipline… first rotation assigned was obstetrics…on that first night in obstetrics he was on call and he called the friend who was not on call…he was in tears saying he did not know what to do, he’d been sent into the delivery room to take care of deliveries…and he had never even seen a lady with her clothing off and here he was expected to do a delivery.”  (Physician 1)

Lack of exposure to the lesbian, gay, bisexual, and transgendered patient population was also noted. Cultural differences toward mental health issues and lack of prior exposure to patients with mental health conditions were perceived as challenges for some IMG residents, in terms of illness recognition and treatment modalities.  Their previous medical training abroad did not include conditions such as depression, anxiety, or stress as these were not considered illnesses in more traditional cultures. 

“…in a lot of countries there are diseases that don’t exist…depression doesn’t exist. Many of the mental health issues don’t exist, addictions aren’t there. And it’s not that they’re not there, but people will never bring them up as an issue, so if someone’s depressed they never would agree that it’s a medical concern and they’d never be treated for it … There’s no anxiety disorder, there’s no panic attacks, there’s no depression, there’s no alcoholism. There’s no suicide, even though there’s lots of people that commit suicide, it’s all accidents.” (Physician 7)

Participants also noted that some IMG residents struggled with the names and types of diagnostic tests, procedures and medications commonly used in Canada, as these often differed from those in other countries. 

“I came from a culture where medications are prescribed based on generic names.  Coming here it’s brand names, that’s really confusing, although recently there’s been a change to generic, and I think that is so helpful because I think no matter what part of the world, if we all deal with generic names we all know what we’re talking about.”  (Resident 3)

#### Learning challenges

Study participants indicated that various degrees of discomfort was encountered when attempting to move some IMG residents beyond their comfort level in clinical care and explore different approaches to learning.  Particular reference was made to the uneasiness encountered when encouraging residents to develop competencies related to critical thinking skills, areas of clinical and procedural deficiencies, and developing an understanding approach to learning. Many were previously trained in education systems that emphasized rote learning and memorization, rather than self-directed learning and critical thinking.

“A big challenge is that the physicians have often been trained in very different learning environments than Canadian graduates, so often they’ve been taught a lot of…. memorization and much less around critical thinking. …I often spend a lot of time trying to get international grads to tell me how they got to a diagnosis, rather than focusing on working from the diagnosis onward...” (Physician 9)

It was perceived that those educated in hierarchical societies were more likely to expect their supervisor to have all the answers and appeared unwilling to question or challenge their preceptor/supervisor.  Moreover, they seemed somewhat uncomfortable and uneasy with receiving feedback on their performance and may have felt challenged by it.

“The style of many medical training systems abroad is…so hierarchical that you end up not daring to either question what is said by someone who is teaching or ask a question of someone that’s teaching you, and you’re expected just to watch and not touch. Canadian medical culture has over the last 30 or 40 years evolved away from that for the large part…” (Physician 1)

“…so to receive any negative feedback whatsoever is extremely difficult and often met with defensiveness because they feel like if I did poorly on something and I gave you negative feedback that you’re going to fail me…I’m not accepting that feedback because if I accept that feedback I’ll fail.” (Physician 7)

Participants remarked that IMG residents appeared to have only superficial knowledge of Canada’s publicly administered health care system and navigation through the complexities of the acute care, long-term care, and community-based sectors of the health care system was a challenge. Many were also noted to lack knowledge and skills in computer and information technology, specifically electronic medical records.    

“Challenges right from something that’s basic as even using the electronic medical record system.  It’s not something we do.  We’re more into paper charting.” (Resident 3)

Participants recalled instances when some IMG residents were guided by other paradigms of ethical and legal norms.  In particular, challenges with patient confidentiality and privacy, informed consent, and professional honesty emerged as issues. Preceptors understood that IMG residents came from a different cultural environment and one of the challenges was helping IMGs understand the meaning of Canadian ethical principles.

“…from a legal and technical point of view [there] have been instances where there wasn’t an understanding of what confidentiality was…the major one being sending in references that attached to them were named patient records.” (Physician 1)

“They don’t recognize the grey areas affecting it [ethical decision making] cause they don’t recognize it requires informed consent, informed decision making. It requires confidentiality, it requires understanding the person’s values and wishes, it requires understanding their cultural background, their religious background.” (Physician 3)

Participants noted that at times they felt they were not being told the truth and that the whole notion of saving face seemed more important to some IMG residents than the collegial attempt to get at the truth.

“…to us honesty is number one; you have to tell the truth; but in a lot of cultures, no; loyalty to family and friends is more important. …That’s just a value of what’s most important and [saving] face is more important than truth.” (Physician 6)

Those who were trained in physician-centric hierarchical systems were perceived to experience some challenges in Canada’s patient-centered care environment where patients themselves expect to make informed decisions about the care they receive. The role of the physician is then to provide the necessary information so that patients can make informed decisions about their care, rather than the physician making the decision for the patient. These IMG residents were then challenged with clearly communicating and explaining treatment options to the patient, building a trusting doctor-patient relationship, reaching common ground, and dealing with noncompliance, all of which takes time.  The expectation that patients would unquestionably accept what the doctor said was challenged.

“…it’s particularly difficult for international graduates that have come from hierarchical systems where some of the doctors have been the ones making the decisions and the patients usually defer to them because the doctors are the ones with the expertise and good judgment.” (Physician 3)

#### Cultural differences

Participants commented on cultural issues of gender inequality, specifically related to the subordinate position of females.  The perception was that some male IMG residents from cultures with prevailing socially constructed gender roles were at times dismissive toward female supervisors/colleagues or preceptors.

“They’ve challenged me personally being a female preceptor so they tend to walk over you a bit, and you have to stand your ground and push back and just remind them about the gender equality.” (Physician 3)

Within the primary care setting, some IMG residents were observed to experience challenges with working in multidisciplinary teams.  Those who were accustomed to hierarchical and vertical working relationships were perceived to be less efficient in their work. The less formal, more collaborative and horizontal working relationship prevalent within the Canadian context appeared to be a challenge in that it necessitated relationship building with individuals, rather than issuing orders. 

“…Residents who have come from [some countries]...men especially would come over with high expectations. They wouldn’t say anything to me. They would totally almost ignore me, and it took probably a good year for them to realize that women had status and that if they wanted to get anything done that they would have to talk to me.” (Allied Health Professional 9)

Cultural differences in gender roles and gender equality were repeatedly noted as being a challenge for those IMG residents from countries with prevailing differences in socially constructed gender roles.  While greeting patients with a hand shake is the socially acceptable norm in Canadian society, some cultures do not permit non-essential touching with members of the opposite sex, except for family members.  This was identified as being an issue for some patients and health professionals in western society. 

“…we have people who will not shake hands with patients, so male physicians who won’t shake hands with women, women physicians who won’t shake hands with males.” (Physician 6)

Participants remarked that some IMG residents unknowingly encroached upon one’s personal or individual space, in that they came too close in physical proximity to another individual during a conversation.  This encroachment of personal space was perceived to be psychologically uncomfortable and appeared to breach Canadian norms of keeping an appropriate physical distance between individuals during interaction. 

"…after about 3 days I had to take him aside and said, you [want to] spot off two feet and you do not cross those two feet, otherwise I can’t have you in my clinic because you’re intimidating my patients.  Because he would go to here, he liked to speak from 6 inches. …once he realized, he said really, I do that?  Oh OK.  And it was quite amusing, at time, I would see him watching the floor tiles." (Physician 2)

It was perceived that IMG residents at times disregarded boundary issues related to gift-giving in the workplace.  They were seen as stepping outside the limits of professional boundaries when giving substantive gifts to their colleagues, supervisors/preceptors. Such gift-giving was usually interpreted as a request for a favour in return sometime in the future. Canadian recipients usually felt uncomfortable with receiving the gifts and declined more substantive gifts.

“But these guys are always bringing you personal gifts. It's one of the things that we noticed, and it's hard for us, and they don't understand why we just kind [of] take a step back...clothing and jewellery." (Allied Health Professional 3)

Some IMG residents were noted to experience difficulties in understanding and interacting with Canada’s culturally diverse patient population.  Participants noted that it would be beneficial to develop cultural competency skills in interacting with patients from distinctive cultural groups with differing cultural norms, values, and beliefs, so as not to unconsciously impose their own cultural values on patients. 

 “…if they’re from an area of the world where the population’s really homogeneous they’re not used to the multicultural mosaic that is Canada.” (Resident 1)

#### Personal struggles

Participants fully recognized and were empathetic to the many personal struggles faced by IMG residents.  The stresses endured throughout the immigration process and relocation to a country with different systems and cultural norms and a vastly different climate were considered to be immense.  Most immigrant IMGs were older, married with children, faced financial pressures, and did not have an extended familial support system in Canada.  The arduous process of writing Canadian exams and applying for a residency position was demanding, time consuming, and strenuous.

“…the hoops that you have to jump through to get back [into practice], it’s incredible. I admire and I can’t quite comprehend how people with children manage to do it. It’s such a huge financial toll in terms of not just the exams, but most people have to do a lot of volunteer work or [get] paid very minimally in order to get the experience; the reference letters, and things that they need, and to get a reputation in the community that they’re applying for a residency program, for example, in.” (Resident 4)

## Discussion

Our study exploring the cultural transition of immigrant IMG family medicine residents into Canadian medical culture reveals that IMGs bring multiple strengths to Canadian practice including strong clinical knowledge and experience, high education level, the richness of varied cultural perspectives, and positive personal strengths.  These strengths are perceived to add value to patient care in Canada’s multicultural society.  At the same time, IMG family medicine residents appear to experience challenges in the areas of communication and language, clinical practice, learning styles, cultural differences, and personal challenges.  These challenges can affect both the doctor-patient relationship and the preceptor-resident relationship.   

Communication skills and English language proficiency, both verbal and written, were identified repeatedly by physicians and health professionals as a major challenge for IMG residents.  These findings are consistent with other Canadian studies,^11^^,^^12^ as well as with US patients’ perceptions of communication skills of IMGs.^13^  In family practice, effective communication skills are key to patient interviewing and history taking, establishing the doctor-patient relationship, negotiating a treatment plan, and communicating with other health professionals. Communication may be lost or misunderstood when language nuances and body language are not picked up. The safety and quality of patient care may also be affected if written chart orders are difficult to read.  Moreover, difficulties with English language skills may be misinterpreted as being slow or lacking in medical skills.  The findings point to a gap in formal language and communications training in IMG orientation programs and/or residency training programs.  

The finding that some IMG residents did not recognize language and communication as being an issue is of some concern in that communication is central to the doctor-patient relationship in the Canadian context.  If IMGs are sensitive to language issues, then discussing communication concerns with them may evoke defensiveness and may be perceived as discriminatory.  A previous study found that IMGs tend to perceive ethnicity, culture, or language to be the basis of discrimination.^14^ It is also possible that IMG residents did not identify communication and language as challenges due to perceived pressure to perform and not wanting to show weaknesses.  The importance of effective communication skills in family practice in Canada necessitates that residency training programs approach the issue with sensitivity.

A specific communication issue identified as a challenge for some IMGs was eye contact. In western culture, eye contact is deemed to be a central element of communication, as it is considered important in opening and closing a conversation and signifying interest, attention and understanding in what is being communicated, as well as in building trust between individuals. Lack of eye contact during conversation may be interpreted as being uninterested, distracted, disrespectful, or lacking in self-confidence.  While a range of socially appropriate boundaries exist related to eye contact within each culture, cultural and religious differences prevail with respect to the appropriateness and duration of eye contact in communication.  It is possible that IMG residents modulate eye contact based on cultural awareness, while those from western cultures may be lacking in such awareness. For IMGs from certain cultures, adjusting to direct eye contact may be particularly difficult because it challenges a deeply engrained cultural norm; education programs, health professionals and patients should recognize this.

Gender issues also appear to prevail across several themes for IMGs transitioning into Canadian medical culture, including communication, areas of clinical deficiencies, and learning styles. The social norm in some cultures regarding non-essential touching (e.g. handshaking) with members of the opposite sex can be readily accommodated within the Canadian health care setting, however, not providing needed care to patients of the opposite sex is professionally unacceptable in Canadian society.  In Canada, discrimination on the basis of gender is prohibited under the law.^15^ Preceptors should be aware that some IMG residents may not have had any medical exposure to patients of the opposite sex, therefore, may require training in the area before starting a particular rotation (e.g. obstetrics/gynecology). Family medicine residency programs are mandated to train all residents in the full range of clinical core competencies and skills for all genders and across the full lifecycle of patients.  Not providing care to patients of a particular gender based on the provider’s religious or cultural beliefs and norms is not an option in Canada. 

A further gender-related issue that was identified was the authority of female preceptors.  It was felt that their position of authority as a teacher was often not respected by male residents who came from societies with strictly defined gender roles.  This becomes problematic in establishing the preceptor-learner relationship. It may be that these residents are missing out on a form of support, guidance and professional role modeling that the female preceptor can provide. Having an open attitude can foster an effective preceptor-learner relationship. 

Given that differences in medical education and learning styles exist, particularly between western and other countries,^16^ it is not surprising that IMG residents were noted to experience learning challenges within the Canadian context. Medical education in Canada is based on principles of adult self-directed learning, is problem-based, focuses on the development of critical thinking skills, and emphasizes collaboration and relative equality between teacher and learner.  In contrast, medical education in other cultures has been described as hierarchical, subject-oriented, lecture-focused, and teacher-centered.^17^ Preceptors should recognize that IMGs trained in some other cultures may find it difficult to challenge their preceptors, ask questions, or accept feedback, as it may be their perception that the teacher’s role is to provide the answer and the learner’s role is to memorize it.  IMG residents will also need to work toward developing skills in self-directed learning. Preceptors should also understand that what may be considered as constructive feedback by Canadian standards, can be interpreted as personal criticism and failure by IMG residents.^18^ Establishing and maintaining open and positive communication between the resident and preceptor and explaining to the resident the differences in educational styles are ways to mitigate negative perceptions of feedback.

Cultural differences in ethical norms were evident in our study.  IMG residents may well experience some tensions between their own ethical convictions related to confidentiality, informed consent, and professional honesty and the principles and values of medical ethics in Canada.  This points to the need for specific focused training in Canadian medical ethics.  The Canadian Medical Association Code of Ethics^19^ outlines the guidelines for an ethical framework for practicing physicians.   

For some IMG residents, lack of exposure to mental health conditions due to differing cultural perspectives was identified as an area of clinical deficiency.  In some cultures, mental illness is stigmatized and brings shame on the family; as such it remains mostly hidden, undiagnosed, and untreated.  In such circumstances, there is limited physician exposure to patients with mental health issues.  Preceptors should be aware of this limitation and focus the IMG resident experience on developing competencies in the area of mental health.

Consistent with another study,^11^ some IMG residents were noted to experience challenges working in a collaborative team-based model with other health professionals.  It is possible that the roles of various health care professionals in the team were unfamiliar to the IMGs, therefore, hindering the appropriate use of team members.  Also, differences in scope of practice for physicians and other health professionals from different countries may result in confusion among IMG residents regarding which tasks are typically performed by which health care provider.^20^^,^^21^ The hierarchical medical structures from countries of origin of some IMG residents place physicians on top, with the expectation of possessing comprehensive knowledge.  As such, IMG residents may be reluctant to ask for help from or consult with other professionals.  

Adopting a more egalitarian relationship between the patient and physician and including the patient as an active member of the team who can make his/her own decisions about care was identified as a challenge for some IMG residents.  Rao^22^ notes that in collectivistic cultures, such as Asia or Africa, the patient-doctor relationship is more paternalistic and authoritarian, rather than collaborative and egalitarian, as in western cultures. For physicians integrating into western culture, this discordance can result in difficulties in establishing and maintaining an effective relationship with patients.  In family medicine residency training in Canada, teaching and role modeling of the doctor-patient relationship should emphasize its egalitarian aspect.

We are confident that a theoretical level of data saturation has been reached in this study and in the quality of the study data. This is evidenced by the rich, thick description provided by study participants, the in-depth data collection arising from one hour long interviews/focus groups, and the meaningful coherence of the findings which emerged from the analysis of the study data.

Our study has several limitations.  The qualitative study data are based on the perceptions of physicians and other health professionals who train and work with IMG family medicine residents.  A more robust study of cultural integration issues would include direct observation over time.  The perceptions of the study participants were based on their recalled observations of and experience with many IMG residents over time and participant recall bias may have tended in the direction of challenges rather than strengths. Our study did not assess the progression or evolution of IMGs becoming culturally competent over time. A shortcoming of the study is the small number of IMG resident participants.  We anticipate that there may be differences in responses between IMG residents and their preceptors and the health professionals who work with them.  It is not known if the study findings apply to IMGs who enter medical practice in Canada through routes other than a family medicine residency program or to IMG residents in other specialty training programs.

Further research in the area of communication competencies and language sensitivities of IMG residents, as well as studies employing a larger sample of IMG residents examining their experiences with the cultural transition into Canadian medical practice, are warranted.

## Conclusions

This study furthers the understanding of the cultural transition of IMG residents into family practice in Canada and contributes to what is currently a small body of literature on IMGs within the Canadian context. Challenges appear to occur in the areas of communication, gender role and gender interaction differences, patient-centered approach to care, recognition of mental health illness, ethical issues, knowledge of the health care system, and learning styles.   Both residency training programs and IMGs themselves have a role to play in addressing these challenges.Residency programs must recognize the challenges that can occur during the cultural transition to Canadian family practice and incorporate Canadian medico-cultural education into the curriculum.  IMG residents also need to be aware of the cultural differences that may exist and be open to different perspectives and new ways of learning.  The study findings may resonate with immigrant physicians practicing in other parts of the world, as well as highlight some of the cultural challenges that physicians planning to immigrate to Canada may experience when transitioning into medical practice.

### Acknowledgments

This study was funded by a Janus Research Grant sponsored by the Research and Education Foundation of the College of Family Physicians of Canada.  Ms. Rebecca Leonard conducted a review of the literature for this manuscript.

### Conflict of Interest

The authors declare that they have no conflict of interest.
